# Evaluation of a Rapid Test for the Diagnosis of Cholera in the Absence of a Gold Standard

**DOI:** 10.1371/journal.pone.0037360

**Published:** 2012-05-30

**Authors:** Anne-Laure Page, Kathryn P. Alberti, Vital Mondonge, Jean Rauzier, Marie-Laure Quilici, Philippe J. Guerin

**Affiliations:** 1 Epicentre, Paris, France; 2 Ministry of Health, Kinshasa, Democratic Republic of Congo; 3 Institut Pasteur, Unité des Bactéries Pathogènes Entériques, Centre National de Référence des Vibrions et du Choléra, Paris, France; 4 Nuffield Department of Clinical Medicine, Centre for Tropical Medicine, Centre for Clinical Vaccinology and Tropical Medicine (CCVTM), University of Oxford, Oxford, United Kingdom; Aga Khan University, Pakistan

## Abstract

**Background:**

Early detection and confirmation of cholera outbreaks are crucial for rapid implementation of control measures. Because cholera frequently affects regions with limited laboratory resources, rapid diagnostic tests (RDT) designed for field conditions are important to enhance rapid response. Stool culture remains the “gold standard” for cholera diagnosis; however, its lack of sensitivity may lead to underestimation of test specificity. We evaluated the Crystal VC® immunochromatographic test (Span Diagnostics, India) for cholera diagnosis using a modified reference standard that combines culture-dependent and independent assays, or a Bayesian latent class model (LCM) analysis.

**Methodology/Principal Findings:**

The study was conducted during a cholera epidemic in 2008, in Lubumbashi, Democratic Republic of Congo. Stools collected from 296 patients were used to perform the RDT on site and sent to Institut Pasteur, Paris, for bacterial culture. In comparison with culture as the gold standard, the RDT showed good sensitivity (92.2%; 95% CI: 86.8%–95.9%) but poor specificity when used by a trained laboratory technician (70.6%; 95% CI: 60.7%–79.2%) or by clinicians with no specific test training (60.4%, 95% CI: 50.2%–70.0%). The specificity of the test performed by the laboratory technician increased to 88.6% (95% CI: 78.7–94.9) when PCR was combined with culture results as the reference standard, and to 85.0% (95% CI: 70.4–99.2), when the Bayesian LCM analysis was used for performance evaluation. In both cases, the sensitivity remained high.

**Conclusion:**

Using an improved reference standard or appropriate statistical methods for diagnostic test evaluations in the absence of a gold standard, we report better performance of the Crystal VC® RDT than previously published. Our results confirm that this test can be used for early outbreak detection or epidemiological surveillance, key components of efficient global cholera control. Our analysis also highlights the importance of improving evaluations of RDT when no reliable gold standard is available.

## Introduction

In May 2011, the World Health Assembly recognized the re-emergence of cholera as a significant global public health problem. In recent years, the incidence of cholera has been increasing regularly, with approximately 317 000 cases and 7500 deaths reported by the World Health Organization (WHO) in 2010, representing an increase of 43% in the number of cases and 52% in the number of deaths as compared to 2009 [1]. The major outbreak in Haiti contributed in large part to this increase, but epidemics of varying sizes also occurred in many other areas of the world, with 48 countries reporting cholera cases and 32 countries reporting deaths in 2010.

Early outbreak detection and confirmation is crucial for the rapid implementation of appropriate interventions. Whereas culture is required for confirmation as well as for characterization of the outbreak strain, rapid diagnostic tests (RDT) probably represent the most promising tools for early detection in areas without laboratory resources.

One of the most recent cholera RDTs available on the market is the Crystal VC® RDT (Span Diagnostics Ltd, Surat, India), a dipstick assay initially developed by the Institut Pasteur [2], [3]. The test is based on the detection of the lipopolysaccharide of *Vibrio cholerae* O1 and O139 by monoclonal antibodies and uses a one-step, vertical-flow immunochromatography principle and colloidal gold particles-conjugated antibodies for detection of bound antigens [3].

To date, published studies on the test prototype developed and produced by the Institut Pasteur or on the commercial version showed high sensitivity, ranging from 92% to 100% [3]–[5]. Initial evaluations of the prototype on frozen stool samples with known etiology showed specificities ranging from 84% to 100% [3]. However, subsequent prospective evaluations of both the test prototype or the commercial version, carried out during cholera epidemics or in endemic settings, consistently showed lower specificities ranging from 71% to 77% when used on bulk stool [4]–[7]. Higher specificities of 92%–95% were obtained when the test was used on enriched rectal swabs [2], [3], [5]. One study showed that specificity was also affected by the skill level of the user, with specificities of 67% and 76% when the test was performed by field clinicians or laboratory technicians, respectively [4].

In most of these evaluations, stool culture is used as the reference standard for estimating performance. Although it remains the reference method for laboratory surveillance of cholera, stool culture cannot be considered a perfect gold standard as it lacks sensitivity [8].

Any evaluation against a reference standard with low sensitivity leads to underestimation of the specificity. To address this problem, a combination of techniques can be used to improve the reference standard —most commonly culture together with PCR. Although the use of PCR on stool specimens to detect DNA targets specific to *V. cholerae* O1 or O139 is not validated as a gold standard for cholera diagnosis, its theoretical ability to detect low numbers of organisms or dead cells suggests that it could improve the sensitivity of a new reference standard. Alternatively, statistical approaches using latent class models (LCM) and Bayesian inference approaches have been applied to estimate test performance in the absence of a gold standard [8]–[11]. The Bayesian LCM combines prior hypotheses on test characteristics with actual observations to estimate the performance of each test included in the evaluation. In this study, we used both of these approaches to evaluate the performance of the Crystal VC® RDT during a cholera outbreak in the city of Lubumbashi, Democratic Republic of Congo (DRC).

## Methods

### Ethics

Ethical approval was obtained from the Ethics Committee of the Ecole de Santé Publique, Kinshasa, DRC and the “Comité de Protection des Personnes”, Ile de France XI, France. The study was conducted according to the principles expressed in the Declaration of Helsinki. Written informed consent was obtained from all study participants or for minors, from their parents or legal guardians.

### Study population

The study took place in two cholera treatment centers (CTC) supported by the non-governmental medical organization Médecins Sans Frontières (MSF) in Lubumbashi, DRC. Over 3500 cholera cases were reported in the city between October 2007 and May 2008. The study started in March 2008, towards the end of the outbreak. Patients presenting to the CTC were included in the study if they were over 5 years of age, had acute watery diarrhea with or without vomiting, and if they, or their guardian, signed a written informed consent form. Exclusion criteria were declared ingestion of antibiotics in the previous 7 days and/or absence of stool during the observation period.

Sample size calculation was based on an expected sensitivity of 95% and a specificity of 80%. For 5% and 6% precision, respectively, and with an alpha risk of 5%, 73 confirmed positive and 171 confirmed negative cases were needed. In cholera treatment centers, patients were classified according to their dehydration status, based on WHO criteria [12]. Based on expert opinions, we estimated that the prevalence of cholera was very high (∼100%) among patients with severe dehydration, high (∼70%) among patients with some dehydration and moderate (∼50%) among patients with no signs of dehydration. According to these estimates and in order to represent all dehydration stages, we calculated a sample size of 421 patients stratified as follows: 32 patients with severe dehydration, 53 patients with some dehydration and 320 patients with no dehydration.

The sample size planned to test inter-batch reliability was 163 samples, based on the following hypotheses and parameters: expected kappa coefficient of 0.9, a precision of 7%, an alpha risk of 5% and a proportion of invalid results of 5%.

### Rapid diagnostic test

For each patient included in the study, a stool sample was collected and used to perform two Crystal VC® tests, one by a trained laboratory technician, and a second by a nurse or medical doctor working in the CTC but untrained in the use of the test (together they are referred to as the “clinicians”). To evaluate inter-batch reliability, the laboratory technician tested a subset of samples with two lots of RDT.

The laboratory technician performed the test according to the manufacturer's instructions for use, after training at Institut Pasteur. The only explanation provided to clinicians was a French translation of the manufacturer's instructions. This ensured that conditions for the evaluation were similar to those expected in the context of an outbreak. Approximately 200 µl of fresh liquid stool were transferred to a test tube and a dipstick was placed in the tube and left for 15 minutes. Results were interpreted according to the manufacturer's instructions. If the control line did not appear, irrespective of other lines, the test was considered invalid and repeated once.

### Specimen shipment and bacterial culture

Samples were packaged for shipment using two means of transport: (i) in Cary-Blair medium, following manufacturer's recommendations (COPAN Diagnostics, Italia) (ii) on a filter paper disc, dipped into fresh stool and placed into a microtube with 2 to 3 drops of normal saline solution (NaCl 0.9%) [13]. Both transport media were kept at room temperature and sent weekly to Institut Pasteur, Paris, following International Air Transport Association regulations for infectious substances.

Isolation of choleragenic vibrios (*Vibrio cholerae* serogroup O1 or O139) was performed by culture following enrichment steps, according to standard methods [14]. Bacteriological cultures, regarded as the reference test, were carried out blind to RDT results.

### PCR analysis

PCR was used to resolve discrepant RDT and culture results. Due to the fact that the RDT is based on *V. cholerae* O1 or O139 lipopolysaccharide detection, we chose to target the genes specific for the O1 or O139 antigen biosynthesis located in the *rfb* region of the *V. cholerae* chromosome. Culture-negative specimens and a random sub-sample of 27 culture-positive specimens were subjected to examination for detection of *rfb* O1 and O139 sequences by a duplex PCR assay as described by Hoshino *et al.* [15]. For each of the samples tested, one mL of the first alkaline peptone water (APW) enrichment broth obtained from each stool sample and stored at −20°C was later submitted to total DNA extraction. Two extractions methods were used, the InstaGene Matrix (Biorad, France), according to the manufacturer's instructions, or the conventional phenol-chloroform DNA extraction, followed by ethanol precipitation [16]. PCR amplification of 16S RNA encoding genes was used to control for the presence of PCR inhibitors. An additional inhibition control was performed on samples testing negative by PCR for *rfb*O1, by adding a known concentration of positive DNA from our *V. cholerae* O1 reference strain to the extracts.

### Statistical analysis

Data were double entered into EpiData 3.0 software (The EpiData Association, Odense, Denmark) and analyzed using Stata 9.0 (Stata Corporation, College Station, Texas, USA) and WinBUGS [17] for the Bayesian analysis.

### Estimation of sensitivity and specificity using a reference standard

The following definitions were used for the analysis using a reference standard.

Culture reference standard: a sample was considered positive by culture if *V. cholerae* O1 or O139 was isolated from either of the transport media. A sample was considered negative if culture from both Cary-Blair and filter paper were negative for *V. cholerae* O1 or O139. If only one culture result was available (ie. Cary Blair or filter paper sample missing), and this result was negative, the culture result was considered indeterminate and the specimen was excluded from the analysis of RDT performance.

Culture and PCR reference standard: a sample was considered positive if any of the culture or PCR results were positive for the detection of *V. cholerae* O1 or O139. A sample was considered negative if both culture results were available and both negative, and PCR was also negative. As above, specimens with only one negative culture result available were considered indeterminate and excluded from the analysis.

Sensitivity and specificity were measured as the proportion of RDT-positive specimens among positive specimens by the reference standard, and RDT-negative specimens among reference-standard negative specimens, respectively, and the exact binomial 95% confidence intervals were determined. Likelihood ratios were calculated using the following formulas:
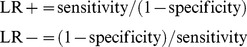
Inter-batch reproducibility was estimated using the kappa coefficient.

### Estimation of sensitivity and specificity using the Bayesian latent class model

We used a Bayesian LCM to estimate the sensitivity and the specificity of the RDT and culture in the absence of a gold standard, as described by Branscum *et al.* [8]. The latent class analysis allows the characterization of a discrete latent class – here the true disease status - by discrete observed variables – culture and RDT results. In this model, both tests are equally considered as imperfect. The Bayesian inference approach using LCM allows the combination of prior information on the test characteristics, described as a prior distribution, with information obtained through observed data to give posterior distribution of the test characteristics.

Prior distributions can be estimated based on a review of the literature and/or expert opinion in the absence of data. Published evaluations of the RDT indicated good sensitivity (92% to 100%) and variable levels of specificity (67% to 100%). These evaluations used culture as the reference standard, which, considering the imperfect sensitivity of culture, might have led to underestimation of the specificity, while sensitivity might be quite accurate. To reflect these hypotheses, we used prior distributions for the RDT characteristics that were uniform over an interval which included previously estimated values: uniform distribution between 0.8 and 1 for sensitivity, and between 0.5 and 1 for specificity.

We considered that culture was 100% specific. Only one publication gave information on culture sensitivity [18]. In this article, 135 suspected cholera cases were investigated by culture, PCR, direct fluorescence microscopy, and RDT. Culture was positive in 86 while 131 specimens were found positive by at least one of these methods, giving a putative sensitivity of culture of 66% [18]. We made a hypothesis for the culture sensitivity of a uniform distribution between 0.6 and 0.9. Finally, the prior distribution of prevalence was considered uniform between 0.5 and 1.

Convergence was assessed by running multiple chains from dispersed starting values [19]. The influence of priors on the estimated model parameters was assessed by successive use of different hypotheses for culture sensitivity. The two tests used here rely on different biological attributes: the presence of live bacteria for culture and antigens for the RDT. As recommended by Branscum *et al.* [8], in the main analysis the tests were considered conditionally independent. To evaluate whether there may be some correlation between the tests depending on bacterial load, we also assessed the influence of adding a conditional correlation between the tests. The Deviance Information Criterion (DIC) was used to compare the models.

## Results

### Patient characteristics

The study started on March 2^nd^, 2008 and ended prematurely on May 2^nd^ the same year, when weekly cases dropped below 5 and MSF ended its intervention. During this period, 296 patients were included in the study, with a median age of 29 (IQR 18–41) and sex ratio M∶F of 1.21. Signs of dehydration were severe in 51 (17%) patients, moderate in 73 (25%), and absent in 172 (58%). This distribution was not representative of all patients presenting to the CTC, since inclusions were deliberately selected to include patients presenting with different dehydration states, including no dehydration.

### RDT results

Using the RDT on site, the laboratory technician reported 192 positive results for O1, one positive for *V. cholerae* O1+O139, and 103 negative results. The clinicians reported 167 *V. cholerae* O1 positive results, 24 *V. cholerae* O139 positive results, 10 *V. cholerae* O1+O139 positive results, 91 negative and 4 indeterminate results. Since we observed that untrained users had difficulties in differentiating the O1 and O139 lines, all positive results for O1 and/or O139 were considered as O1 positive in the analysis. The inter-batch correlation tested by the laboratory technician on 117 samples was very good (kappa = 0.96; CI 95% 0.78–1.00).

### Culture results

The median delay between sample collection and inoculation in Paris was 13 days (range 7–17 days). Culture results were obtained for 256 patients, and indeterminate in 40. Culture was positive in 154 patients and negative in 102. All *V. cholerae* isolates found in this study were *V. cholerae* O1 serotype Inaba.

### PCR results

PCR using the phenol-chloroform extract as a template gave a positive signal for amplification of 16S RNA encoding genes for a sub-sample of 60 specimens tested, while the InstaGene method extracts gave only 80% 16S RNA-PCR positive specimens, suggesting the presence of substances inhibitory to the PCR assay. The phenol-chloroform extracts were used for the rest of the analysis.

All 27 culture-positive specimens tested were positive by PCR. One culture-negative specimen was lost and could not be tested retrospectively. Among the 101 culture-negative specimens tested by PCR, 47 were RDT positive (by the laboratory technician, the clinician, or both) and 54 RDT negative; 32 showed a positive PCR signal for the *rfb* O1 gene, all of which were also positive by the RDT. None of the samples was positive for *V. cholerae* O139 by PCR.

All samples giving negative results by PCR were additionally tested for detecting inhibitors under the strict conditions of the *rfb* PCR assay, by adding 1 µl of target DNA in the reaction. A positive signal was observed in all samples.

### Performance of RDT using culture or culture and PCR as the reference standard

Using the culture results described above as the gold standard, the RDT showed good sensitivity, but poor specificity, resulting in a low positive likelihood ratio (Tables 1 and 2). The training of the user had no impact on the test sensitivity. Specificity was lower in the untrained user group (clinicians), although the difference was not statistically significant.

**Table 1:** Cross-tabulation of the RDT results performed by the laboratory technician or by clinicians compared to the reference standards culture, and culture and/or PCR.

		Culture		Culture and/or PCR		
		Positive	Negative	Positive	Negative	Total
Laboratory technician	Positive	142	30	164	8	172
	Negative	12	72	22	62	84
Clinicians	Positive	122	31	171	12	153
	Negative	32	70	15	57	102
	Indeterminate	0	1	0	1	1
	Total	154	102	186	70	256

**Table 2:** Performance of the rapid test for the diagnosis of cholera, according to the skill level of the user, N = 256.

User	Sensitivity % (95% CI)	Specificity % (95% CI)	LR+ (95% CI)	LR− (95% CI)
Reference standard: culture				
Laboratory technician	92.2 (86.8–95.9)	70.6 (60.7–79.2)	3.14 (2.31–4.25)	0.11 (0.06–2.19)
Clinician	92.9 (87.6–96.4)	60.4 (50.2–70.0)	2.45 (1.85–3.25)	0.26 (0.18–0.38)
Reference standard: culture or PCR				
Laboratory technician	88.2 (82.6–92.4)	88.6 (78.7–94.9)	7.72 (4.01–14.84)	0.13 (0.09–0.20)
Clinician	91.9 (87.0–95.4)	82.6 (71.6–90.7)	5.29 (3.16–8.86)	0.10 (0.06–0.16)
Bayesian analysis				
Laboratory technician	93.0 (88.3–96.6)	85.3 (69.8–99.2)	34.15 (3.06–119.69)	0.16 (0.01–0.33)
Clinician	93.8 (89.2–97.2)	78.4 (59.6–98.7)	23.90 (2.31–69.66)	0.23 (0.01–0.43)

When the PCR results were included in the reference standard, the RDT sensitivity fell moderately while specificity rose to levels above 80% for both the laboratory technician and clinicians (Table 2).

### Performance of the RDT using a Bayesian LCM

The Bayesian LCM assuming conditional independence between culture and RDT resulted in a sensitivity of 93.0%, similar to the sensitivity found in the analysis using culture as a gold standard. Specificity was 85.0% when the test was performed by a laboratory technician and 78.4% when the test was performed by a clinician (Table 2).

As the performance of culture for the detection of cholera is not well known, sensitivity of the statistical method was assessed using several hypotheses for culture sensitivity. The sensitivity analysis was also done using a model for conditionally dependent tests. Using a model for independent tests, the estimated sensitivity of RDT remained the same regardless of the hypotheses of culture sensitivity, while the estimated sensitivity of culture and specificity of the RDT varied in opposite directions (Table 3). Introducing a hypothesis of conditional dependency between culture and RDT decreased the estimates of RDT sensitivity and specificity. Specificity remained higher than estimated using culture as the gold standard, with values ranging from 77.4% to 91.0%, and several estimates around 85%. The DIC of the two models (with and without conditional dependency) were similar, indicating that both models were similarly adequate to match the data.

**Table 3:** Posterior distributions according to the different hypotheses on culture sensitivity.

	Culture		RDT				
Hypotheses	Sensitivity		Sensitivity		Specificity		DIC
	%	95% CI	%	95% CI	%	95% CI	
Independent tests							
60–100%	83.3	[70.2–98.0]	93.0	[88.2–96.6]	81.8	[65.0–98.8]	27.01
60–90%	79.9	[69.8–89.4]	93.0	[88.3–96.6]	85.3	[69.8–99.2]	27.22
60–80%	75.5	[68.8–79.8]	93.0	[88.3–96.6]	91.5	[78.6–99.6]	27.06
Dependent tests							
60–100%	79.4	[64.2–97.3]	87.7	[80.4–95.1]	78.5	[60.0–98.6]	26.9
60–90%	77.3	[64.5–89.2]	87.5	[80.4–95.1]	80.1	[60.4–98.8]	27.02
60–80%	72.6	[63.2–79.7]	86.8	[80.3–94.7]	85.1	[65.6–99.2]	26.98

## Discussion

An imperfect reference standard, i.e. culture, is an often-cited limitation in evaluations of rapid diagnostic tests for cholera. To date, the only alternative proposed is to investigate discordant results using PCR [6], [7]. Several PCR methods, targeting various genes, have been suggested but there is no current consensus on a validated PCR method for cholera diagnosis, especially regarding pre-treatment of stool specimens for PCR assay. We chose to use the PCR assay proposed by Hoshino *et al.* as it was specific for O1 and O139 LPS of *V. cholerae* [15], which is also detected by the RDT. Our results suggest that this multiplex PCR is more sensitive than culture. As a consequence, the estimates of RDT performance using a composite reference standard of culture-dependent and independent are substantially different from estimates using only culture as the reference standard. While the sensitivity was only slightly reduced, the specificity was increased substantially, from 70% to 88% for the RDT performed by a trained laboratory technician.

Interestingly, the results obtained using a statistical method specifically designed for evaluations in the absence of a gold standard were very similar to the estimates using the improved reference standard combining culture and PCR. For all hypotheses of culture sensitivity we considered, the Bayesian LCM analysis resulted in a comparable sensitivity, above 90% and an increased specificity, above 80%. The culture sensitivity, which was also modeled as a parameter of this analysis, ranged between 72% and 84%. We believe that this approach allows a more accurate estimate of true test performance.

Previous evaluations of the test prototype developed by Institut Pasteur [4], [5] and recent evaluations of Crystal VC® in India [6], [7] have also shown low specificities compared to culture as a gold standard. We were able to apply the Bayesian LCM analysis to the results of the study by Wang *et al.* [5], and found that the specificity of the test used on bulk stool increased from 77% to 90% using our main hypotheses for prior distributions. Similarly, results from Mukherjee *et al.* [7], which show a specificity of 72.9% compared to culture as a gold standard, are consistent with a specificity of 94.2% using Bayesian LCM analysis. The corresponding culture sensitivities were 76.4% and 67.2%. We suggest that the results of future studies be analyzed using the Bayesian approach to account for imperfections in the gold standard, especially if the RDT is compared to culture only. This could potentially have an important impact on the outcome of the evaluation and on the future use of the test.

Several factors should be taken into account for an optimal use of Bayesian LCM in the future. First, the sample size should be calculated specifically for a Bayesian LCM, as described by Dendukuri *et al.* [20]. This would result in higher sample sizes than for an evaluation comparing to a reference standard, and the desired precision of the estimate should be balanced with the feasibility of the study. Secondly, as a Bayesian analysis relies on prior hypotheses about the different diagnostic methods used, better knowledge and/or expert agreement on the performance of culture for cholera diagnosis would help refine the prior hypotheses and solidify the results. Culture sensitivity can be affected by different parameters, including initial bacterial load, experience of the technician, prior administration of antimicrobial treatment, sample storage conditions and delay between sample collection and inoculation. In our study, highly experienced technicians performed culture, and patients who had taken antibiotics in the week prior to inclusion were excluded from the study. However, specimens were inoculated 7 to 17 days after collection, which may have reduced the sensitivity of culture.

In addition to the limitation that our study was not initially designed for a Bayesian analysis, this evaluation had several shortfalls. First, our sample size was smaller than initially calculated, since the outbreak and MSF intervention ended before the sample size could be reached, and some specimens were excluded for technical reasons. A reduced sample size leads to wider confidence intervals. Although the confidence intervals obtained here are quite wide, we consider the results and conclusions to still be meaningful. Second, we cannot exclude that other PCR assays, such as real-time PCR, could have been more sensitive than the assay used here. Were that the case, the RDT performances would only be considered as stronger. It would be useful to have a formal evaluation of different PCR methods for cholera in order to establish a recognized method for diagnosis and/or evaluation purposes.

### Conclusion

Despite the poor performance of the test in previous evaluations, the Crystal VC® RDT is widely used for the detection of outbreaks, confirmation of cases in case-control studies or other epidemiological uses. Here we show that the test specificity is higher than previously reported, probably due to an imperfect gold standard. Rapid diagnostic tests remain of little added value over clinical assessment for case management during a declared cholera outbreak, but these new results suggest that the test can be used with some confidence for epidemiological purposes. To improve evaluations of rapid diagnostic tests, future studies should use improved reference standards or, if not possible, take into account the moderate sensitivity of culture in the analysis.
